# Influence of MLC901 Alone and with Moderate Exercise on Pain Response Concurrent Due to Stress of Male Mice

**DOI:** 10.31661/gmj.v8i0.1253

**Published:** 2019-07-10

**Authors:** Maryam Nasehi, Farshad Ghazalian, Nader Shakeri, Mohammad Nasehi, Mohammad-Reza Zarrindast

**Affiliations:** ^1^Department of physical education and sport sciences, Islamic Azad University, Science and Research Branch, Tehran, Iran; ^2^Cognitive and Neuroscience Research Center (CNRC), Tehran Medical Sciences Branch, Islamic Azad University, Tehran, Iran; ^3^Institute for Cognitive Science Studies (ICSS), Tehran, Iran

**Keywords:** Exercise, Stress, MLC901, Pain, Mice

## Abstract

**Background:**

Physical exercise is known to have a positive effect on pain responses induced by stress, while chronic stress causes a negative effect on cognitive abilities. Depending on the type, duration, and intensity of the stressor, it can induce analgesia or hyperalgesia. Furthermore, the beneficial effects of traditional Chinese medicine MLC901 on stress processes have been reported. Here, the effects of MLC901 and moderate physical activity on pain response in restraint-stressed mice was investigated.

**Materials and Methods:**

Male NMRI mice were used in this study and were restrained in plexiglass mesh restrainers for induction of chronic restraint stress. Treadmill exercise was carried out for moderated exercise, 5 days/week for 4 weeks. MLC901 was intraperitoneally administered in the experimental groups. The pain response of the adult NMRI mice was detected via the hot-plate test.

**Results:**

It was showed that intraperitoneal administration of MLC901 dose (0.4 but not 0.1 and 0.2 mg/kg; once/2 days; for 25 days) resulted in the decreased percentage of time in the hot plate, indicating hyperalgesia. Moreover, restraint stress for 3 but not 6 and 9 hours/day elicit hyperalgesia in mice. The data showed that subthreshold dose of MLC901 (0.1 mg/kg) reduced hyperalgesia in 3-day stressed mice. Moderate treadmill running (10 meters/min for 30 min/day, 5 days/ week) potentiated the effect of 6 and 9 days on pain (induced hyperalgesia) that was blocked by MLC901 (0.1 mg/kg).

**Conclusion:**

Our findings indicated that subthreshold dose of MLC901 alone or when it associated with moderate exercise decreased hyperalgesia induced by stress, indicating the protective effect of MLC901.

## Introduction

Stress is mostly defined as a feeling of being worried, overloaded, and tense. Stress-dependent disorders have been widely known recently. Stress has been connected to other diseases such as cancer, coronary heart stroke, and poor mental health. Although well performance and task famishment are results of low stress, high stress can be harmful and can intervene in our normal life. Pain suppression (stress-induced analgesia [SIA]) or exacerbation (stress-induced hyperalgesia [SIH]) are results of stress depending on the intensity, duration, and nature of the stressor [1]. Yilmaz *et al*. (2010) defined SIA as a reduced pain response after stress exposure and SIH as an increased pain response after stress exposure, which is mediated by reducing or increasing pain-inhibitory circuits and may be an indicator of adequate centrally mediated pain control [2]. Brain-derived neurotrophic factor (BDNF) plays a critical role in improving cognitive factors and reducing stress in individuals, and increasing BDNF increases brain resistance to neuronal degeneration and brain damage. Previous studies have shown that stress reduces neurogenesis and the level of the hippocampus of BDNF, leading to cognitive impairment and increased stress in individuals [3]. Some studies mentioned that widespread changes to the neurochemical, neurobiological, and behavioral responses of the brain are caused by stress. Stress also affects neural plasticity negatively by producing a deficiency in learning and memory processes [4, 5]. Results of the study by Watanabe *et al*. (1992) showed that rodents exposed to chronic stress for weeks or months displayed reduced dendritic complexity within the hippocampus [6]. It was also found that complexity within the hippocampus is typically correlated with impaired spatial memory, a hippocampal function, and various pain responses [6]. There are several ways in which acute and chronic exercise may help to decrease stress and its physiological and psychological responses [7]. Firstly and from a cognitive aspect, being more physically active can enhance self-concept, tolerance, resilience, physical self-perceptions and perceived energy, and decrease fatigue. Above mentioned properties cognitively impel the ways we evaluate intrinsic and extrinsic event of various positions [7]. If engagement in acute exercise involves social interaction with people, it can help distraction away from rumination about stressors. Secondly, from a physiological aspect, our physical response to a psychological stressor (stress reactivity) can be improved by exercise. Moreover, attenuated physiological responses (e.g., heart rate, blood pressure, etc.) can also be enhanced by physical activity [7]. Previous research examined the role of exercise on learning, memory, and stress on both humans [8, 9] and animal models [10, 11]. Many researches showed that exercise led to the improvement of functional recovery in patients with Parkinson [12] or Alzheimer [13]; such improvement is a result of exercise on brain function, local neurogenesis and long-term potentiation (LTP) in the hippocampus [14, 15]. Many studies have emphasized that running exercise plays a fundamental role in the enhancement of chronic stress-induced memoryshortage [11, 16, 17]. Kim and Leem [18] reported that exercise impacts on incensement of hippocampal AMP-activated protein kinase-mediated BDNF that it can protect chronic stress-induced memory impairment [18]. MLC901 (NeuroAid II), a traditional medicine utilized in China for patients after stroke has been formerly reported to impel neuroprotection and neuroplasticity [19]. It has been suggested that MLC901 activates K_ATP_ channels [20] ad induces the protein kinase B survival pathway [21], several crucial mechanisms involved in neuroprotection [22]. It leads to increased neurogenesis [19], increased expression of the cortical BDNF, and more brain neuronal activity [23]. Cognitive effects of MLC901 in normal, stress mice and exercise mice were investigated. The obtained data showed that MLC901 leads to the reduction of hyperalgesia in stress mice and improves the implementation of mice in novel object recognition. Besides, some researchers proposed a promising therapeutic potential for MLC901 [24]. Above interesting results led us to evaluate the effect of an ineffective dose of MLC901 alone or with a moderate treadmill running on pain response concurrent due to the stress of male mice.

## Material and Methods

### 1. Animals

One hundred and sixty Male NMRI mice (weighing 24-28 g) from a breeding colony at the Institute of Cognitive Science (Tehran, Iran) were utilized. Standard conditions (a 12-h light/dark cycle with lights on from 07:00–19:00 h, and controlled temperature 22 ± 2° C) was used. Separate cages were also used for them (10 per cage). A week before the onset of the actual experiment, mice were freely fed and got familiarized with the experimenter and the testing environment. All procedures were carried out according to the Guide for the Care and Use of Laboratory Animals (approval code: N.R.C., 2011).

### 2. Restraint Stress Model

In chronic restraint stress experiments, mice were restrained in plexiglass mesh restrainers, which prevented forward or backward movement, 3 h/day for 3، 6, and 9 days. Mice were returned to their own cages, after restraining treatment.

### 3. Treadmill Running

Treadmill exercise was carried out at 10 meters/min for 30 min/day, 5 days/week for 4 weeks. All mice pre-exercised to habituate them to treadmill running (Borj Sanat, Iran) at 10 meters/min for 20 min/day, for 5 days. Sedentary mice were placed on the treadmill that was turned off for 30 min once a day.

### 4. Hot-Plate For Evaluating Pain

Eddy and Leimbach in 1953, proposed the hot plate test, which is a test of the pain response in animals. It is utilized in examining the impact of analgesics by observing the reaction to pain resulted by heart and basic pain research [25]. Using the hot-plate test baseline, nociceptive sensitivity was examined. The machine has a 25 ×25 cm metal hot-plate surface set at 51°C, a plexiglass cage that fits over the hot-plate and a foot-switch timer. One of the indications of pain threshold is a delay to lifting wrinkles. An average of three trials was performed for hot-plate latency with 10min intervals between each trial. Between trails, the testing apparatus was thoroughly cleaned [26].

### 5. Drug Treatment

MLC901 composed of nine herbal components including the following composition per capsule: 0.16 g *Rhizoma acori tatarinowii*, 0.16 g *Radix paeoniaerubra*, 0.16 g *R. chuanxiong*, 0.16 g *R. angelicae sinensis*, 0.16 g *Carthamus tinctorius*, 0.16 g *Prunus persica*, 0.16 g *R. salvia miltiorrhizae*, 0.16 g *R. polygalae*, and 0.80 g *R. astragali*. MLC901 batch BN112 provided by Moleac (Singapore) was used for all experiment. MLC901 was intraperitoneally administered at dose of 0.1, 0.2, and 0.4 mg/kg according to a pilot study. Volume 10 ml/kg was given to the control group.

### 6. Experimental Design

There were eight mice in each experimental group. Each animal was tested once. The experiment was finished in 30 days, and on the 31^st^ day, the hot-plate test was done.

#### 6.1. Experiment 1

This experiment testified the impact of intraperitoneal MLC901 administration of pain response concurrent due to the stress of male mice. Four groups of non-exercised animals (sedentary treadmill mice) got various doses of MLC901 (0, 0.1, 0.2, and 0.4 mg/kg) for once/48h during 30 days (from 8^th^ day to 32^nd^ day). The hot-plate test was carried out on the day after the last MLC901 administration.

#### 6.2. Experiment 2

In experiment 2, mice were separated into four sets of four groups as follows: Restraint stressed groups: to investigate effect of different periods of restraint stress on pain response concurrent due to stress of male mice, four groups of non-exercised animals were subjected to 0, 3, 6, and 9 consecutive days of restraint stress with duration 3 h/ day. Hot-plate test was performed one day after the stress exposure period. MLC901 combined with restraint stress to investigate the effect of an ineffective dose of MLC901 administration on pain response concurrent due to stress of male mice. Four groups of non-exercised animals were manipulated with MLC901 at a dose of 0.2 mg/kg and then subjected to restraint stress in various days with the same stress protocol. Then, the hot-plate test was done on the 31^st^ day. Treadmill running combined with restraint stress: to investigate the effect of moderate physical exercise on pain response concurrent due to stress of male mice, four groups of animals were placed to the treadmill and subjected to restraint stress in various days with the same stress protocol. Then, the hot-plate test was done on the 31^st^ day. MLC901 in association with treadmill running with restraint stress: to investigate the effect of ineffective dose of MLC901 and treadmill running on pain response concurrent due to stress of male mice, four groups of animals were administered with MLC901 and then placed to the treadmill and subjected to restraint stress in various days in last days. Then, the hot-plate test was done on the 33^rd^ day.

### 7. Statistical Analysis

Data were analyzed via SPSS 19 (IBM Company, U.S.A.) using one- or two-way ANOVA, and Tukey post-hoc tests. All values are reported as mean ± standard error (SE). Statistical significance was set at P<0.05.

## Results

### Effect of MLC901 Administration On Pain of Male Mice

**Figure 1:**
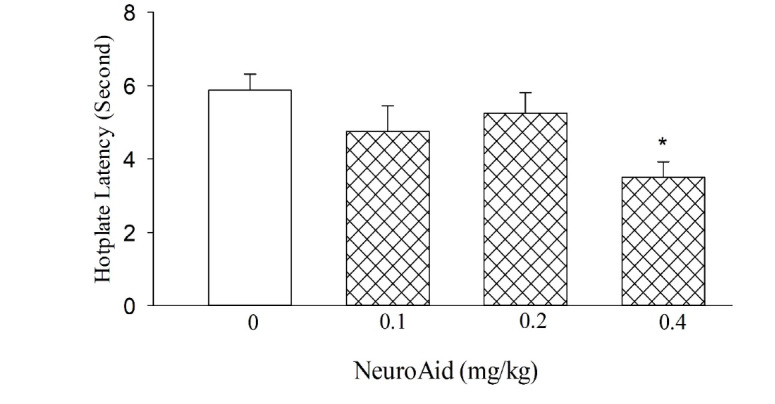
The effect of different NeuroAid dosages on pain response. A long-term foot lift (hot-plate test) of mice receiving different amounts (0.1, 0.2, and 0.4 mg/kg NeuroAid). There was a significant difference between the control group on the test day. *P<0.05

As shown in Figure-1 MLC901 at doses of 0.1 and 0.2 mg/kg did not alter percentage of time in hot plate, the drug at dose of 0.4 mg/kg resulted in the decreased percentage of time in the hot plate, indicating hyperalgesia (F [3,28]=3.449, P=0.03; Figure-1) compared to control group.

### Effect of Stress On Pain Response to Stress Male Mice

**Figure 2:**
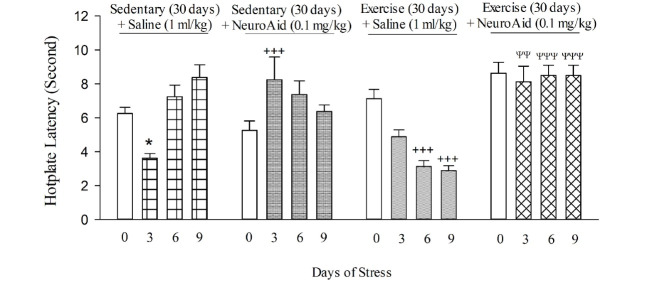
Effect of different stress in present and absence of NeuroAid effect, exercise, and co-current of treatment between NeuroAid +exercise on pain perception. *P<0.05 vs. control group; ^+^P<0.05, ^++^P<0.01, and ^+++^P<0.001) vs. control group; ^Ψ^P<0.05, ^ΨΨ^P<0.01, and ^ΨΨΨ^P<0.001 vs. control group.

As shown in Figure-2, the exposure to restraint for 3 but 6 and 9 days resulted in the decreased percentage of time in hot plate stress (F [3,28]=13.406, P≤0.001; Figure-2). In conclusion, 3-day stress resulted in hyperalgesia but 6 and 9 days stress did not hyperalgesia in comparison with the control group.

### Effect of MLC901 On Responses Induced by Stress On Pain

As shown in Figure-2, there was a significant interaction between MLC901 and the exposure to restraint stress on percentage of time in the hot plate (stress effect: F [3,28]=13.406, P≤0.001; Figure-2), and stress-MLC901 interaction (F [3,56]=8.342, P≤0.01). Moreover, MLC901 at dose of 0.1 mg/kg decreased hyperalgesia induced by 3-day stress in mice.

### Effect of Moderate Treadmill Running On Responses Induced by Stress On Pain

As shown in Figure-2, there was a significant interaction between treadmill running and various days of restraint stress on hot plate (restraint stress effect: F [3,56]=8.631, P≤0.01; treadmill running effect: F [1,56]=29.647, P>0.005; restraint stress-treadmill running interaction: F [3,56]=24.969, P≤0.001). The data showed that treadmill running decreased percentage of time 6 and 9 days restraint-stressed mice in the hot plate, indicating moderate treadmill running resulted in hyperalgesia 6 and 9 days restraint-stressed.

### Interaction Between MLC901 with Moderate Treadmill Running and Various Days of Restraint Stress in Hot-Plate

As shown in Figure-2, there was an interaction between various days of restraint stress and combination of MLC901 plus treadmill exercise on percentage of time in the hot plate (restraint stress: F [3,56]=9.162; P=0.01; combination of MLC901 and treadmill running effect: F [1,56]=111.897, P≤0.01; restraint stress-combination of MLC901 and treadmill running interaction: F [3,56]=4.435, P=0.07). Moreover, the data showed that a subthreshold dose of MLC901 reduced hyperalgesia-induced by co-current treatment between exercise and stress.

## Discussion

This study aimed to investigate whether MLC901 have a protective role in the effect of moderate treadmill running on hyperalgesia concurrent due to the stress of male. The main finding of the present study was that chronic restraint stress or 3-days stress resulted in hyperalgesia but 6 and 9 days stress did not hyperalgesia in comparison with the control group. Hyperalgesia in the hot-plate and that harmful effect of chronic restraint stress cannot be prevented by moderate treadmill running, but MLC901 can help to moderate physical activity for prevention deleterious effect of stress on hyperalgesia in the mice. Our results indicate that only the 3-days restraint stress decreased the percentage of time in the hot plate and resulted to hyperalgesia, but some previous studies have demonstrated painless concurrent due to stress of male mice [27-29]. However, other studies show chronic SIH and them similar to our study [30, 31]. Our results indicate that chronic administration of MLC901, at applied doses in this study, resulted in decreased hyperalgesia of 3 days stress in mice. Previous studies have demonstrated the beneficial effect of MLC901 in rodent model of stroke (focal ischemia), cardiac arrest (global ischemia), traumatic brain injury or normal rodents [19, 21, 24]. This study is the first attempt to evaluate the effect of MLC901 in the stressed mouse model. It has been demonstrated that MLC901 stimulates hippocampal neurogenesis and promotes differentiation of newborn cells in neurons [19, 21, 23]. This property can explain the effect of MLC901 on flexible cognitive processing. The data show that only the exposure to 3 days restraint stress decreased the percentage of time in the hot plate and ineffective dose of MLC901 could restore this impairment. Substantial evidence implicates that chronic restraint stress reduces hippocampal neurogenesis and mRNA levels of BDNF and induces hippocampus-dependent cognitive deficits [18, 32]. On the other hand, MLC901 increases BDNF expression and causes proliferation of cells which differentiate and mature into neurons [33]. It is likely that chronic pretreatment with MLC901 via increased BDNF expression prevents the impairing effect of restraint stress. In this study, besides MLC901, treadmill exercise resulted in hyperalgesia 6 and 9 days in mice. Previous studies showed that in the forced exercise model (running on a treadmill by electric shock), the axis of the adrenal-pituitary-hypothalamus is activated and increases the level of adrenal steroids and increased stress in the mice while continuous running is not a stressor [34]. In our study, moderate treadmill running produced hyperalgesia in stressed mice. Regarding these findings, we examined whether an ineffective dose of MLC901 can potentiate the protective effect of treadmill running on hyperalgesia in stressed mice. The results obtained from this experiment show a positive answer to this question. MLC901 potentiate the protective effect of treadmill running on retrieval of hyperalgesia in 3-days stressed mice. Meanwhile, the combination of MLC901 administration and treadmill running decreased pain in 6- and 9-days restraint-stressed mice. It is concluded that MLC901 can use as a therapeutic option for hyperalgesia induced by stress. Also, it supports the protective effect of moderate intensity exercise on hyperalgesia in stress conditions.

## Conclusion

The findings indicated that co-treatment of MLC901 with moderated exercise decease hyperalgesia induced by stress, indicating the protective effect of MLC901.

## Acknowledgment

The authors of this paper appreciate and acknowledge the animal neuroscience lab of the Institute for Cognitive Science Studies (ICSS).

## Conflict of Interest

The authors declare there is no conflict of interests.
